# Breast cancer in pregnancy: concurrent cesarean section, nipple-sparing mastectomy, and immediate breast reconstruction—case report

**DOI:** 10.3389/fonc.2023.1332862

**Published:** 2024-01-08

**Authors:** Alessandro Innocenti, Pietro Susini, Luca Grimaldi, Tommaso Susini

**Affiliations:** ^1^ Plastic and Reconstructive Microsurgery, Careggi University Hospital, Florence, Italy; ^2^ Plastic Surgery Unit, Department of Medicine, Surgery and Neuroscience, University of Siena, Siena, Italy; ^3^ Breast Unit, Gynecology Section, Department of Health Sciences, University of Florence, Florence, Italy

**Keywords:** pregnancy-associated breast cancer, breast cancer, nipple-sparing mastectomy, immediate breast reconstruction, case report

## Abstract

**Background:**

Pregnancy-associated breast cancer (PABC), with an incidence rate from 1:3,000 to 1:10,000 deliveries, is the most frequent cancer during pregnancy. PABC appropriate management must take into consideration both the maternal oncological safety and the fetal health, thus posing a challenge for the mother, the baby, and the clinicians. The treatment should adhere as closely as possible to the breast cancer (BC) guidelines. Therefore, surgery is a mainstay, and, when mastectomy is required, breast reconstruction (BR) is a topic of debate. To minimize the risks to the baby, most surgeons postpone BR to delivery. However, a delayed breast reconstruction (DBR) could affect the outcome. In the present case, we report cesarean section concurrent with mastectomy and immediate breast reconstruction (IBR).

**Methods:**

A 37-year-old patient, at the 36th week of pregnancy with PABC, underwent simultaneous cesarean delivery, nipple-sparing mastectomy, and IBR. To minimize risks for the newborn, cesarean was firstly performed under spinal anesthesia. Immediately after, breast surgery, including mastectomy and IBR, was performed under general anesthesia. Partial submuscular IBR with an acellular porcine dermal matrix concluded the surgical procedure. Lactation was inhibited, and adjuvant chemotherapy and hormone therapy were administered to the patient.

**Results:**

In a single surgical session, cesarean delivery, subcutaneous mastectomy, axillary dissection, and IBR were successfully carried out. No early or late postoperative complications were reported for both the patient and the newborn. Histopathological investigation reported a multifocal and multicentric infiltrating ductal carcinoma. After a 6-year follow-up, the patient is alive and well.

**Conclusion:**

To the best of our knowledge, this is the first reported case of concomitant cesarean delivery, PABC mastectomy, axillary dissection, and IBR. This surgical strategy allowed PABC treatment by the BC guideline, minimizing the newborn’s disadvantage and permitting, at the same time, the best final BR outcome.

## Introduction

Pregnancy-associated breast cancer (PABC) is defined as breast cancer (BC) diagnosed during pregnancy or up to 1 year after delivery (1). Despite its relatively low incidence, ranging from 1:3,000 to 1:10,000 pregnancies, it represents the most common cancer in pregnancy (2, 3). Due to the breast physiological changes occurring in pregnancy, PABC could pose a severe diagnostic challenge (3, 4). Moreover, psychological and ethical aspects play a crucial role in PABC because the appropriate management must fulfill both the oncological threat and the pregnancy (5–7).

According to the most accredited guidelines, BC surgical treatment during pregnancy should be as close as possible to the standard treatment of non-pregnant patients. Because immediate breast reconstruction (IBR) currently represents one of the most popular reconstructive methods, when possible, it should be considered even in PABC (8–10).

Although BC surgery is commonly performed during all trimesters of pregnancy (11), timing and method for BR are largely debated in the literature. With the aim to minimize the newborn risks, delayed breast reconstruction (DBR) is frequently preferable. However, IBR has other advantages, such as the avoidance of a secondary surgical procedure, the reduction of patient’s distress, and, possibly, more favorable outcomes (12–14).

Although mastectomy and IBR during pregnancy have been already reported (12, 15), to the best of our knowledge, this is the first paper to report concurrent cesarean delivery, PABC mastectomy, axillary dissection, and IBR. Hereby, our team will present the case report, at every stage, and will discuss the potential risks and benefits of this PABC approach pathway.

## Case report

### Clinical case and preoperative evaluation

**Figure 1:**
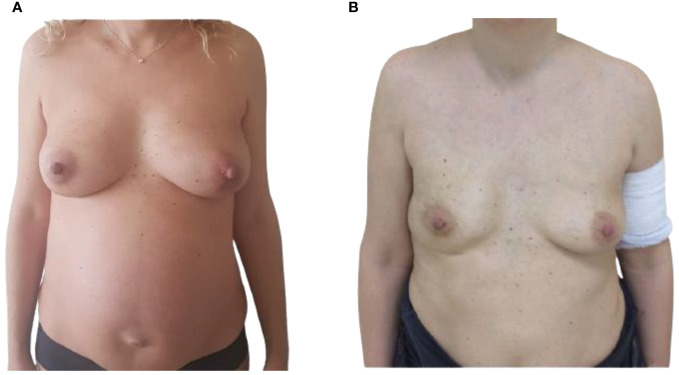
**(A)** Pre-operative frontal view of 37-year-old, 36-week pregnant woman, presenting with a right breast PABC. **(B)** Post-operative frontal view showing the result after 1 year from the right IBR.

The presence of a 7-cm firm mass in the right breast of a 37-year-old patient was confirmed by the ultrasound investigation at the 34th week of pregnancy (**Figure 1**). The patient had no previous relevant medical, family, or psycho-social history. Physical examinations showed a firm nodule in the upper lateral pole of the right breast. Palpation of the axillary lymph nodes was negative for lymphadenopathy. A 14-gauge semi-automated core biopsy (Precisa^®^) allowed the diagnosis of an invasive carcinoma: ER+, 90%; PgR+, 70%; Ki67 index, 25%; and Human epidermal growth factor receptor 2 (HER2)-positive, score 3+.

From the discussion with the patient, her perspective was to protect the fetus, even at the expense of her own health. Following a multidisciplinary approach including breast surgeon, gynecologist, plastic surgeon, oncologist, psychologist, and neonatologist, concomitant cesarean delivery, subcutaneous nipple-sparing mastectomy, sentinel lymph node biopsy, and IBR were planned at 36th week, after induction of fetal lung maturation. The surgical timing considered both the mother and the ongoing pregnancy, avoiding general anesthesia for the fetus, limiting the risks of an excessively premature birth, allowing adequate fetal lung maturation, and ensuring appropriate management of the oncological threat.

### Surgical procedure

**Figure 2:**
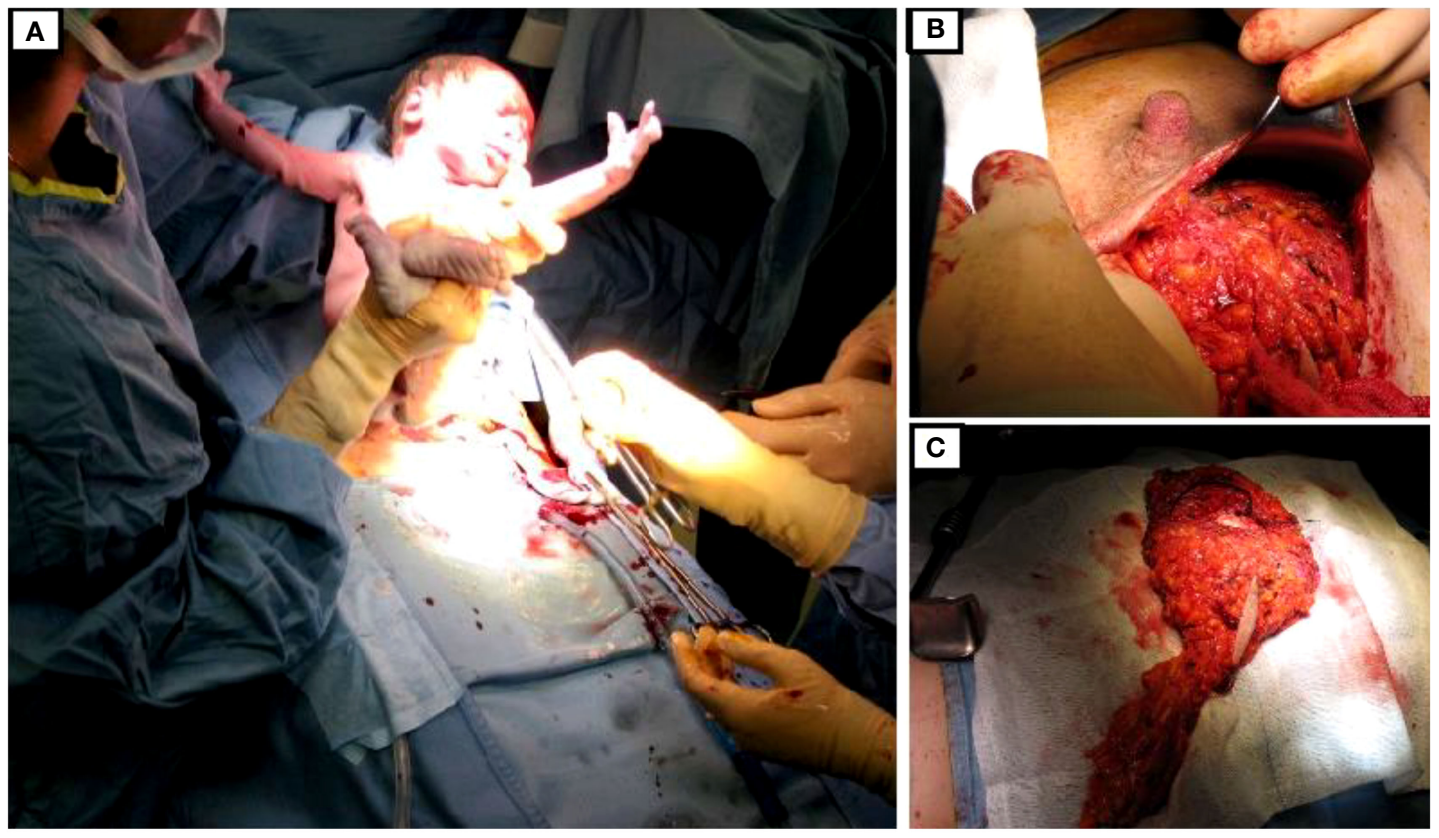
**(A)** The newborn. **(B, C)** Nipple-sparing mastectomy with *en bloc* axillary dissection.

On the second day of the 36th week, cesarean delivery was firstly performed under spinal anesthesia as usual. Fetal monitoring prior to the cesarean delivery was routinely performed by cardiotocography. As soon as extracted, the newborn was taken care of by the neonatologists and was in good health (APGAR index, 9/9/10). Immediately after, general anesthesia was induced, and the patient underwent nipple-sparing mastectomy, sentinel lymph node biopsy with intraoperative frozen section examination, and IBR. In fact, after cesarean section, there were no longer contraindications for general anesthesia, which is routinely adopted for breast oncological procedures. Due to the presence of sentinel lymph node macro-metastases on frozen-section, radical ipsilateral axillary dissection was performed. IBR consisted of partial submuscular coverage of the breast implant by the pectoral major muscle, covering the inferior part of the prosthesis with an acellular porcine dermal matrix. Drains were applied as in routinely authors’ practice (**Figure 2**).

## Results

The whole surgical sequence, including anesthesia times, lasted 240 min. Specifically, the surgical time for cesarean section was 25 min and that of nipple-sparing mastectomy, axillary lymph node dissection, and IBR was 140 min.

The patient was discharged on the fourth postoperative day. Breast drain were removed on the sixth postoperative day when drainage was less than 20 cc, whereas axillary drain on the fourth post-op. No major or minor complications were reported during recovery for both the mother and the newborn.

Pathology report showed a multifocal and multicentric no–special type G3-infiltrating ductal carcinoma with vascular and lymphatic infiltration. Four out of the 18 lymph nodes removed were involved. According to TNM 2017 VIII edition classification, the oncological staging reported the following: pT2 (m) (50mm) and pN2a (4/18). According to American Joint Commettee on Cancer (AJCC) TNM eighth edition stage IIIA, immunohistochemical staining was ER+ of 90%, PgR+ of 90%, Ki67 index of 50%, and HER2-positive score of 3+. No pathologic BRCA1 and BRCA2 mutations or additional abnormal findings were detected at genetic study. No distant metastases were found by skeletal scintigraphy and total body CT investigation.

**Figure 3:**
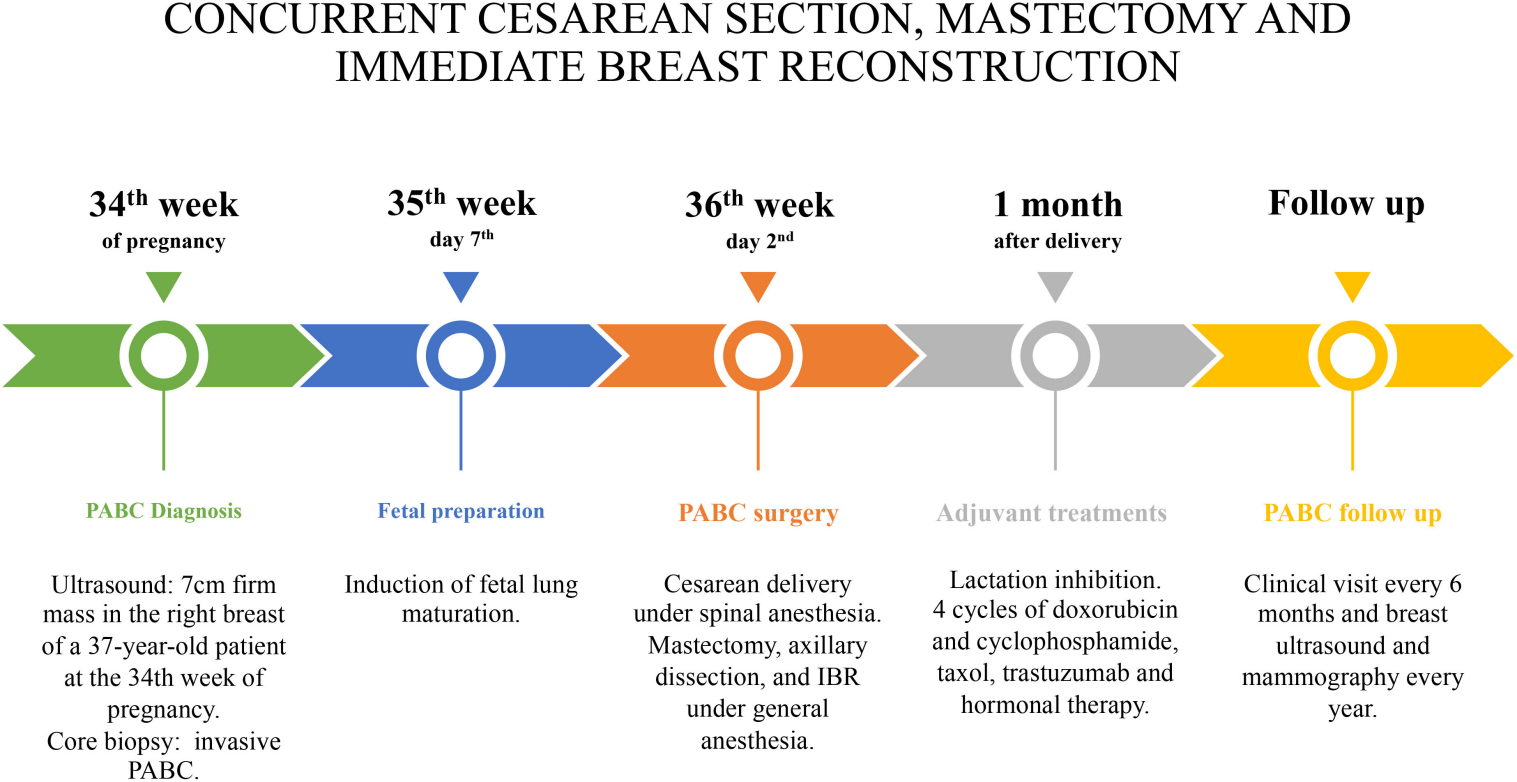
Timeline. PABC, pregnancy-associated breast cancer; IBR, immediate breast reconstruction.

Lactation inhibition was obtained by oral administration of a single dose of 1 mg of cabergoline, because adjuvant chemotherapy was necessary to be administered. The following treatment was started 4 weeks later: four cycles of intravenous AC (doxorubicin and cyclophosphamide) every 3 weeks, followed by 12 taxol weekly administration; trastuzumab every 21 days for 18 months; and hormonal therapy with 3.75 mg of triptorelin 1 fl every 28 days for 3 years and 20 mg of tamoxifen one tablet per day for 2 years, later replaced with 25 mg of exemestane one tablet per day. Radiotherapy (40 + 15 Gy) was administered to the operated breast/chest wall. The follow-up consisted of clinical examination with testing for Ca-15.3 and Carcinoembryonic antigen (CEA) every 6 months and breast ultrasound, mammography, and total body CT scan every year. After a 6-year follow-up, the patient is alive and disease-free (**Figure 3**).

## Discussion

Although 80% of breast lesions during pregnancy are benign (16), PABC occurs in between 1:3,000 and 1:10,000 pregnancies, representing 0.2%–3.8% of all BC (2, 3). Despite its current low incidence, the burden of PABC will probably increase in the next years due to the trend to postpone pregnancy after 40 years that is taking place in many developed countries. In fact, as reported by Robertson et al., older-age pregnancy represents a sensitive risk with an increment of 5.3% per year beyond 25 years (17–19).

Breast modifications that occur during pregnancy and lactation, including hormonal changes, or increased levels of insulin-like growth factor–1, may be related to the enhanced incidence of PABC (20, 21). In addition, pregnancy-related immunological changes such as cellular immunosuppression and immune tolerance may also play a role (22).

Indeed, breast hypertrophy, increase in gland density, and nipple changes typically occur during pregnancy. In addition, the attention of patients and clinicians is more focused on the pregnancy issues. Therefore, PABC diagnosis is often delayed, resulting in many instances in a more advanced and aggressive disease (3, 4).

Although the prognosis of PABC is similar to that of non-pregnant BC of the same stage (3, 23, 24), delayed diagnosis and, consequently, more advanced stage at the onset as well as unfavorable histological features including higher rates of hormone-receptor negative tumors, HER2 overexpression, and lower prevalence of tumor-infiltrating lymphocytes typically occur in PABC. These features account for the worse prognosis of PABC (9, 25–27).

When technically feasible, breast conserving treatment should be considered in the second and third trimester of pregnancy, followed by post-delivery radiotherapy and systemic treatment as appropriate (28, 29). However, mastectomy is always indicated in the first trimester and often required also in the second and third trimester because of multicentric or locally advanced disease. Therefore, BR should be considered in a majority of these cases.

To minimize fetal risks, the 2010 European consensus suggested DBR versus IBR (30). Nevertheless, IBR offers considerable advantages such as a single-step surgical procedure for mastectomy and BR, reduced patients’ distress, minor costs, and shorter waiting lists for the healthcare system. Moreover, due to an easier placement of the breast implant, IBR, respecting the infra mammary fold, optimizes the aesthetical outcome and reduces the need of contra-lateral breast symmetrization procedures.

Lohsiriwat et al. (12) in 2013 firstly proposed IBR during pregnancy, whereas Caragacianu et al. (15) reported the onset of intraoperative uterine contraction requiring tocolysis in one of the 10 patients undergoing IBR. Despite their favorable results, IBR during pregnancy could increase risks including preterm delivery, miscarriage, and fetal distress (31).

In the current case, taking advantage of a diagnosis in the third trimester of pregnancy, the cesarean delivery performed as usual under spinal anesthesia allowed avoidance of the surgical risks for the fetus associated with the breast procedure, including general anesthesia. The planned surgical sequence allowed, at the same time, a single-step procedure, ensuring patients’ comfort, reducing psychological distress and allowing a satisfactory final IBR. Indeed, this approach was made possible by a strong collaboration within the multidisciplinary team of the Breast Unit, including breast surgeon, gynecologist, and plastic surgeon, and especially favored by the setting that, in our hospital, put together the Breast Unit and the Obstetrics and Gynecology Department. In addition, the breast surgeon in this case was also a gynecologist, thus further ensuring the appropriate consideration of both oncologic and obstetric issues.

The main drawback of this surgical strategy is that its applicability is limited to PABC diagnosed in the late third trimester. Indeed, an excessively anticipated preterm cesarean section may expose the fetus to the well-known risks of prematurity, including respiratory distress or even intraventricular hemorrhage. Future research is warranted to confirm the feasibility and safety of this surgical sequence and its appropriate timing.

## Conclusions

PABC poses complex challenges that require a careful balance between appropriate cancer treatment and well-being of the fetus. In the current case, concomitant cesarean delivery, nipple-sparing mastectomy, axillary dissection, and IBR were successfully carried out, without complications for the mother and the baby and with no disease recurrence after a 6-year follow-up. This approach may be considered a suitable treatment for selected PABC cases in the third trimester. Further investigations are necessary to validate this surgical approach.

## Data availability statement

The raw data supporting the conclusions of this article will be made available by the authors, without undue reservation.

## Ethics statement

Ethical approval was not required for the studies involving humans because no experimental practices were performed. The studies were conducted in accordance with the local legislation and institutional requirements. The participants provided their written informed consent to participate in this study. Written informed consent was obtained from the individual(s) for the publication of any potentially identifiable images or data included in this article.

## Author contributions

AI: Conceptualization, Data curation, Formal Analysis, Investigation, Methodology, Supervision, Validation, Visualization, Writing – original draft, Writing – review & editing. PS: Data curation, Formal Analysis, Investigation, Methodology, Validation, Visualization, Writing – original draft, Writing – review & editing. LG: Validation, Visualization, Writing – review & editing. TS: Conceptualization, Data curation, Formal Analysis, Investigation, Methodology, Resources, Supervision, Validation, Visualization, Writing – original draft, Writing – review & editing.
